# Remote interviews for medical residency selection during the initial COVID-19 crisis: a national survey

**DOI:** 10.1186/s12909-021-02890-7

**Published:** 2021-08-31

**Authors:** Mohamad-Hani Temsah, Fadiah Alkhattabi, Fadi Aljamaan, Khalid Alhasan, Adi Alherbish, Mona Philby, Fahad Alsohime, Mohamad Alobaylan, Hayfa Alabdulkarim, Badr Almosned, Deema Gashgarey, Ghadah Felimban, Ziyad Alkathiri, Randa Almaghrabi, Amr Jamal, Mazin Barry, Sami A. Alhaider, Basim Alsaywid, Fahad A. Bashiri

**Affiliations:** 1 Pediatric Department, College of Medicine, King Saud University, PO BOX 2925, Riyadh, 11461 Saudi Arabia; 2 Pediatric Department, King Faisal Specialist Hospital and Research Center, Riyadh, Saudi Arabia; 3 Critical Care Department, College of Medicine, King Saud University, Riyadh, Saudi Arabia; 4 Department of Family and Community Medicine, King Saud University Medical City, Riyadh, Saudi Arabia; 5 Evidence-Based Health Care & Knowledge Translation Research Chair, King Saud University, PO Box 2925, (Internal Code 34), Riyadh, 11461 Saudi Arabia; 6 Division of Infectious Diseases, Department of Internal Medicine, College of Medicine, King Saud University and King Saud University Medical City, Riyadh, Saudi Arabia; 7 Saudi Commission For Healthcare Specialties, Riyadh, Saudi Arabia; 8 College of Medicine, King Saud Bin Abdulaziz University for Health Sciences, Ministry of National Guard,, Jeddah, 11461 Saudi Arabia

**Keywords:** COVID-19, Medical residency, E-interviews, Satisfaction, Survey, Stress, Cost-saving

## Abstract

**Background:**

Medical training programs candidate’s interview is an integral part of the residency matching process. During the coronavirus disease 2019 (COVID-19) pandemic, conducting these interviews was challenging due to infection prevention restrains (social distancing, namely) and travel restrictions. E-interviews were implemented by the Saudi Commission for Healthcare Specialties (SCFHS) since the matching cycle of March 2020 to hold the interviews in a safer virtual environment while maintaining the same matching quality and standards.

**Aim:**

This study was conducted to assess the medical training residency program applicants’ satisfaction, stress, and other perspectives for the (SCFHS) March 2020 Matching-cycle conducted through an urgently implemented E-interviews process.

**Method:**

A cross-sectional, nationwide survey (Additional file 1) was sent to 4153 residency-nominated applicants to the (SCFHS) March 2020 cycle.

**Results:**

Among the 510 candidates who responded, 62.2% applied for medical specialties, 20.2% applied for surgical specialties, and 17.6% applied for critical care and emergency specialties. Most respondents (61.2%) never had previous experience with web-based video conferences. Most respondents (80.2%) used the Zoom application to conduct the current E-interviews, whereas only 15.9% used the FaceTime application. 63.3% of the respondents preferred E-interviews over in-person interviews, and 60.6% rated their experience as very good or excellent. 75.7% of the respondents agreed that all their residency program queries were adequately addressed during the E-interviews. At the same time, 52.2% of them agreed that E-interviews allowed them to represent themselves accurately. 28.2% felt no stress at all with their E-interviews experience, while 41.2% felt little stressed and only 8.2% felt highly stressed. The factors that were independently and inversely associated with applicants’ level of stress with E-interviews experience were their ability to represent themselves during the interviews (*p* = 0.001), cost-savings (*p* < 0.001), their overall rating of the E-interviews quality (*p* = 0.007) and the speed of the internet connection (*p* < 0.006).

**Conclusion:**

Videoconferencing was implemented on an urgent basis during the COVID-19 pandemic in the medical residency application process in Saudi Arabia. It was perceived as an adequate and promising tool to replace in-person interviews in the future. Applicants’ satisfaction was mainly driven by good organization, cost-saving, and their ability to present themselves. Future studies to enhance this experience are warranted.

**Supplementary Information:**

The online version contains supplementary material available at 10.1186/s12909-021-02890-7.

## Background

Interviews for residency programs are crucial in the acceptance process, with the on-site face-to-face interview being integrated into the residency application process a long time ago. In fact, interviews yield several pieces of information regarding the applicants that would not be found in their resumes or personal statement and were a chance for the interviewee to see the institution to which they are applying. Several elements other than the portfolio of the applicants should be considered in which every residency review committee will look for in every applicant; these elements include communication skills, attitude, reasons of interest in the specialty and center, honesty, and problem-solving skills, which are best evaluated through personal interview. Alternatively, on-site in-person interviews have many downsides due to cost, travel plans, and time management for both parties. Applicants who are usually from different cities need to fit up to 10 interviews in their tight schedules in a short period together with their own educational and work duties. The other obstacle is the cost of attending all offered interviews, especially if it requires traveling to several cities [1].

As technology is one of the main enhancers of modern healthcare systems, adopting new trends in technology is crucial to align them and optimize their utilization in healthcare services and medical training [2]. There is a growing need for studies to evaluate the implementation of emerging technology solutions that can disrupt traditional ways of managing healthcare services and training.

The start of the coronavirus disease 2019 (COVID-19) pandemic coincided with the start of application process of the postgraduate medical specialties training programs that require the above-mentioned interview process. When the COVID-19 pandemic was declared by the World Health Organization (WHO) in March 2020, this mandated exploring new dimensions in medical training [3]. The SCFHS is the national regulator of postgraduate medical training that involves application, nomination, interviewing, matching, supervising and assessing training programs, qualifying trainees and setting standards for the practice and development of health professions; issuing registration cards and professional classification certificates for all healthcare practitioners in Saudi Arabia. Facing the challenge of infection prevention practices (social distancing and crowd avoidance) during the pandemic, SCFHS decided to substitute face-to-face interviews with E-interviews to reduce the risk of exposing interviewing staff and applicants, who are both healthcare workers and the potential risk of spreading the disease in different health institutions and its disastrous sequelae.

Every year, thousands of candidates apply for residency training programs in Saudi Arabia under the umbrella of the SCFHS, which nominates candidates to proceed with the interviews for the final matching [4]. Every candidate will have multiple interviews in different centers of the specialty they applied for. Besides the portfolio of the applicants, several elements should be considered by the residency review committee, including the applicant’s communication skills, attitude, reasons of interest in the specialty and center, honesty, and problem-solving skills.

Several studies have been conducted regarding using E-interviews in medical and surgical training programs, but most were individualized for one program and conducted in a single center. Besides, E-interviews were offered as an option or adjunct to the traditional face-to-face interview [5, 6]. Most studies have concluded that E-interviews can be a cost-effective and efficient alternative for on-site in-person interviews [5–8]. However, in a study, applicants for urology residency programs felt that E-interviews are less effective than traditional in-person interviews. In contrast, both the applicants and faculty in this study preferred to have E-interviews as an adjunct only to traditional interviews [9].

Thus, this study was conducted to assess the applicants’ satisfaction (all perspectives and different aspects of satisfaction) with the national (SCFHS) newly introduced E-interview process for medical residency training specialties programs candidates for the Matching-cycle of March 2020 during the COVID-19 pandemic.

## Methods

For the March 2020 SCFHS matching cycle, applicants to residency programs were provided with several E-interviews options as a substitution for face-to-face interviews, based on the preference and agreement of both faculty and applicants, including using Zoom or FaceTime applications or others. The E-interview structure included a description of the applied-for training center and the opportunity for the faculty and the candidates to ask questions to assess each other. Our objective was to assess the applicants’ satisfaction and stress level associated with the use of E-interview system using an anonymous survey on a 5-point Likert scale, the efficacy, and its estimated cost-savings.

### Sample size

The study participants were SCFHS medical specialties applicants for the Matching-cycle of March 2020.

### Inclusion criteria

Residency candidates applying for the SCFHS residency programs (March 2020), interviewed through E-interviews.

### Exclusion criteria

Other interviews, including face-to-face interviews.

### Sampling technique

A non-probability, consecutive sampling technique was implemented, as an invitation to participate in the survey was sent to all 4153 residency applicants to the matching interviews for 2020.

### Survey tool validation

The tool was developed based on a literature review by an expert panel, and a pilot study involving 10 candidates was conducted to validate the tool for clarity and consistency. The questionnaires were sent electronically to the participants within 4 weeks of the E-interviews (April 15–30, 2020), with reminders for non-responders after 2 days.

### Data analysis

The means and standard deviations (SDs) were used to describe continuous variables, and categorical variables were presented as frequencies and percentages. The statistical normality assumption was examined using histograms and the Kolmogorov–Smirnov statistical test, and the statistical equality of the variance assumption was assessed using Levene’s test.

Cronbach’s alpha test of reliability was performed to assess the internal consistency of the Likert-based items characterizing different concepts. Besides, a multiple-response dichotomy analysis was performed to describe the frequencies and percentages of the tick-all-that-applies questions. The overall mean scores of the Perceived Stress Scale and Perceived Satisfaction Scale with videoconferencing were computed by adding the items comprising these concepts and dividing the sum by the number of items for each concept. The chi-square test of association was performed to assess the correlations between categorically measured variables. A multivariate logistic regression analysis was performed to explain the perceived stress of the medical residents during the televised assessment interview they had during the COVID-19 pandemic. Statistical Package for the Social Sciences (version 21; IBM Corp., Armonk, NY, USA) was used for all statistical data analyses, and *p*-values of less than 0.050 were used to denote statistical significance.

### Ethical approval

This study on the urgent utilization of videoconference interviews in the residency application process was in accordance with the postgraduate center recommendations (memo # 9/3/311082), and data were collected after the participants have anonymously provided informed consent. Participation was voluntary and not linked to the applicants’ evaluation. The results of this study will be used as a quality improvement project of the SCFHS, and the approval of the SCFHS’s Institutional Review Board (# 0420-03 exp) was obtained before data collection.

## Results

In total, 4153 applicants for the Saudi medical residency training programs underwent E-interviews. Among the 510 respondents in this survey, 51.4% were male, and 48.6% were female. 62.2% of the respondents applied for medical specialties, 20.2% for surgical specialties, and 17.6% for critical care and emergency specialties (Table 1).

**Table 1:** Descriptive analysis of the medical residents’ demographic and professional characteristics

	Frequency	Percentage
Sex		
Female	248	48.6
Male	262	51.4
Specialty		
Other (please specify)	8	1.6
Pediatrics	148	29
Family medicine	32	6.3
Internal medicine	33	6.5
Surgery	27	5.3
Emergency medicine	39	7.6
Psychiatry	17	3.3
Pharmacological	15	2.9
Genitourinary	3	0.6
Radiology	29	5.7
Pathology	15	2.9
Dentistry	17	3.3
Obstetrics and gynecology	35	6.9
Neurology/surgery	10	2
Critical care	51	10
Dermatology	20	3.9
Ophthalmology	11	2.2

Most respondents (61.2%) indicated that they never had previous experience with web-based videoconferences. (Table 2). The majority of respondents (80.2%) in the current E-interviews used Zoom® to conduct E-interviews, whereas 15.9% used FaceTime®, and 3.9% of the respondents were interviewed using other videoconferencing applications. Most respondents (56.5%) used laptops to conduct the E-interviews, whereas 43.5% used mobile phones or tablets.

**Table 2:** Descriptive analysis of the medical residents’ experience and perceptions about online medical students; evaluation of the videoconference interviews

	Frequency	Percentage
**Prior to this videoconference interview, did you have prior experience with this tool?**		
No, my first time using video conferencing	312	61.2
Yes, I used video conferencing before, but first time to use it for residency interview	173	33.9
Yes, I used video conferencing before, including for residency interview	25	4.9
**The electronic videoconferencing device used**		
PC (laptop)	275	53.9
PC (desktop)	13	2.5
Mobile	222	43.5
**The videoconferencing electronic interface/application used**		
Other (please specify)	20	3.9
Face time	81	15.9
Zoom	409	80.2
**The interview allowed me to represent who I am accurately**		
Strongly disagree	42	8.2
Disagree	99	19.4
Neither agree or disagree	103	20.2
Agree	190	37.3
Strongly agree	76	14.9
**My questions about this residency program were answered**		
Strongly disagree	24	4.7
Disagree	30	5.9
Neither agree or disagree	70	13.7
Agree	258	50.6
Strongly agree	128	25.1
**I feel comfortable ranking King Saud University Medical City based on my interview**		
Strongly disagree	53	10.4
Disagree	55	10.8
Neither agree or disagree	147	28.8
Agree	153	30
Strongly agree	102	20
**Compared to the Face-to-Face interview, how much money did the Video Interview save you (in SR)**		
Less than 100 SR	240	47.1
100–500 SR	88	17.3
More than 500 SR	182	35.7

The applicants felt on average 7.96 on a score out of 11 that they would recommend the E-interviews compared to the traditional interview method as 63.3% of the applicants preferred E-interviews, and 60.6% rated their experience as very good or excellent.

The applicants rated their satisfaction with how E-interview allowed them to accurately represent themselves 3.31 out of 5 (SD = 1.18). Further analyzing their satisfaction level has shown that 37.3% agreed that E-interviews allowed them to represent themselves accurately, 14.9% strongly agreed, while 8.2% strongly disagreed, and 9.4% just disagreed. On the other hand, addressing the applicants’ satisfaction whether their queries about the residency programs and the institutions they applied to were adequately answered: 50.6% agreed that their questions were adequately answered, 25.1% strongly agreed, while 4.7% strongly disagreed, 5.9% just disagreed that their queries were answered adequately (Table 2).

Regarding the applicants’ possible causes of satisfaction with E-interviews over traditional interviews were as follows: first, the preparedness for the event as 47.5% felt just enough information was delivered pre-event, 23.5% felt much information was delivered, while only 6.3% felt very little or little information was delivered. Second, cost-saving as 84.1% believed or strongly believed that it saved them costs (refer to Table 2 for more saving details). Third, applicants’ stress level with the SCFHS E-interviews experience has shown that: 28.2% felt no stress at all, while 41.2% felt little stressed, 8.2% felt highly stressed, and 5.3% felt very stressed.

In terms of E-interviews organization, 60.9% felt it was just or very organized, while 0.8% felt the event was very disorganized. Addressing the length of the E-interview, 74.1% felt it was just the right length of time, while 18.4% felt it was short, 2.4% felt it was long, and 1.4% felt it was very long. In terms of internet quality of the E-interviews, the applicants gave on average a score of 3.76/5 regarding picture quality, 3.66/5 for voice quality, 3.90/5 for time flexibility, and 4.19/5 for place flexibility (Table 3).

**Table 3:** Descriptive analysis of the medical residents’ satisfaction indicators with the online applicants’ evaluation

	Frequency	Percentage %, or score
**How likely is it that you would recommend the video conferencing interviews to a colleague mean (SD) Likert (1–11) rating**		7.95 (1.65)
**With the COVID-19 pandemic, how do you find videoconference interviews compared with in-person interviews?**		
Video Interviews are preferable	323	63.3
Equally preferable	96	18.8
Face-to-face interview is preferable	91	17.8
**How stressed were you during the videoconference interview?**		
Not stressful at all	*144*	*28.2*
Slightly stressful	*210*	*41.2*
Moderately stressful	*87*	*17.1*
Highly stressful	42	8.2
Very stressful	27	5.3
**Videoconference interviews decreased the costs for the candidates: mean (SD) 1–5 Likert rating**		
Strongly disagree	20	3.9
Disagree	13	2.5
Neither agree nor disagree	48	9.4
Agree	202	39.6
Strongly agree	227	44.5
**Overall, how would you rate the event? Mean (SD) 1–5 Likert rating**		3.77 (1.1)
Very poor	18	3.5
Fair	40	7.8
Good	143	28
Very good	154	30.2
Excellent	155	30.4
**How organized was the event? Mean (SD) 1–5 Likert rating**		3.79 (0.88)
Very disorganized	4	0.8
Not organized	25	4.9
Somewhat organized	160	31.4
Just organized	205	40.2
Very organized	116	22.7
**Before the event, how much of the information that you needed did you get? Mean (SD) 1–5 Likert rating**		3.87 (0.86)
Very Little information	4	0.8
Little information	28	5.5
Some of the information	116	22.7
Most of the information	242	47.5
A lot of information	120	23.5
**Was the event length too long, too short, or about right? Mean (SD) 1–5 Likert rating**		2.79 (0.61)
Very short	19	3.7
Short	94	18.4
Just the right length	378	74.1
Long	12	2.4
Very prolonged	7	1.4
**Satisfaction with the interview different aspects (1–5 Likert satisfaction scale)**		
Picture quality		3.76 (0.85)
Voice quality		3.66 (1.03)
Battery/power supply issues		4.14 (0.72)
Your time management flexibility		3.90 (0.98)
Your place (office/hospital/home) flexibility		4.19 (0.82)

Bivariate analysis correlating the applicants’ stress level during the E-interviews with different aspects of their experience has shown a significant inverse correlation between their ability to represent themselves during the E-interview and their stress level, which was the same relation cost saving. The ability of the interview to satisfy their queries in general and to rank or being able to decide on the institution they applied to also correlated inversely with their stress level. While their previous experience with web-based video-conference or the type of platform used for the E-interviews used, voice and picture quality did not correlate significantly with their stress level. (Table 4).

**Table 4:** Bivariate analysis of the applicants’ stress levels with different factors related to their experience with the E-interviews

Factor	Stress during the videoconference interviews		Test statistic	***P***-value
	Low	High		
Sex				
Female	216 (49)	46.4%)	χ2 (1) = 0.162	0.688
Male				
Specialty				
Other (please specify)	8 (1.8)	0	χ2 (16) = 24.10	0.088
Pediatrics	131 (29.7)	17 (24.6)		
Family medicine	28 (6.3)	4 (5.8)		
Internal medicine	27 (6.1)	6 (8.7)		
Surgery	19 (4.3)	8 (8.6)		
Emergency medicine	34 (7.7)	5 (7.2)		
Psychiatry	17 (3.9)	0		
Pharmacological	13 (2.9)	2 (2.9)		
Genitourinary	1 (0.2)	2 (2.9)		
Radiology	21 (4.8)	8 (11.6)		
Pathology	14 (3.2)	1 (1.4)		
Dentistry	15 (3.4)	2 (2.9)		
Obstetrics & Gynecology	32 (7.3)	3 (4.3)		
Neurology/surgery	8 (1.8)	2 (2.9)		
Critical care	45 (10.2)	6 (8.7)		
Dermatology	18 (4.1)	2 (2.9)		
Ophthalmology	10 (2.3)	1 (1.4)		
**Prior to this videoconference interview, did you have prior experience in this tool?**				
No	277 (62.8)	35 (50.7)	χ2 (1) = 3.70	0.055
Yes	164 (37.2)	34 (49.3)		
**The electronic videoconferencing device used**				
PC (laptop)	237 (53.7)	38 (55.1)	χ2 (2) = 0.46	0.795
PC (Desktop)	12 (2.7)	1 (1.4)		
Mobile	192 (43.5)	30 (43.5)		
**The videoconferencing electronic interface/application used**				
Other (please specify)	19 (4.3)	1 (1.4)	χ2 (2) = 4.10	0.129
Face Time	74 (16.8)	7 (10.1)		
Zoom	348 (78.9)	61 (88.4)		
**The interview allowed me to accurately represent myself. Mean (SD) Likert agreement (1–5) scale**	3.46 (1.1)	2.33 (1.31)	t(508) = 7.81	< 0.001
**My questions about this residency program were answered. Mean (SD) Likert agreement (1–5) scale**	3.97 (0.90)	3.12 (1.37)	t(508) = 6.72	< 0.001
**I feel comfortable ranking the hospital based on my interview**	3.48 (1.17)	2.77 (1.31)	t(508) = 4.62	< 0.001
**Compared with in-person interviews, how much money did videoconference interviews save you (in SR)**				
Less than 100 SR	204 (46.3)	36 (52.2)	χ2 (2) = 2.36	0.308
100–500 SR	74 (16.8)	14 (20.3)		
More than 500 SR	163 (37)	19 (27.5)		
**How likely is it that you would recommend videoconference interviews to a colleague? Mean (SD) Likert (1–11) rating**	8.10 (1.61)	7.33 (1.74)	t(5083.39	0.001
**With the COVID19 pandemic, how do you find videoconference interviews compared with in-person interviews?**				
Videoconference interviews are preferable	294 (66.7)	29 (42)	χ2 (2) = 39.9	< 0.001
Equally preferable	87 (19.7)	9 (13)		
Face-to-face interviews are preferable	60 (13.6)	31 (44.9)		
**Videoconference interviews decreased the costs for the candidates. Mean (SD) 1–5 Likert rating**	4.32 (0.78)	3.30 (1.52)	t(73.8) = 5.41	< 0.001
**Overall, how would you rate the event? Mean (SD) 1–5 Likert rating**	3.87 (0.98)	3.10 (1.4)	t(79.2) = 4.63	< 0.001
**How organized was the event? Mean (SD) 1–5 Likert rating**	3.83 (0.84)	3.56 (1.04)	t(82.64) = 2.13	0.036
**Prior to the event, how much of the information that you needed did you get? Mean (SD) 1–5 Likert rating**	3.89 (0.85)	3.75 (0.95)	t(85.8) = 1.20	0.25
**Was the event length too long, too short, or about right? Mean (SD) 1–5 Likert rating**	2.82 (0.55)	2.59 (0.86)	t(76.95) = 2.14	0.036
**Rate your satisfaction with the interview aspects below (1–5 Likert satisfaction scale)**				
Picture quality	3.81 (0.81)	3.47 (1.1)	t(80.4) = 2.41	0.018
Voice quality	3.71 (0.98)	3.38 (1.19)	t(83.33) = 2.18	0.032
Battery/power supply issues	4.14 (0.71)	4.12 (0.81)	t(508) = 0.30	0.774
Your time management flexibility	3.94 (.92)	3.65 (1.28)	t(79.5) = 1.80	0.076
Your place (office/hospital/home) flexibility	4.21 (0.78)	4.02 (1.04)	t(80.44) = 1.41	0.163

The multivariate logistic regression analysis of different factors involved with E-interviews experience and their association with the applicants’ odds of having stressful E-interviews experience revealed a significant inverse correlation with their ability to represent themselves during the interviews (*p* = 0.001), cost savings (*p* < 0.001), their overall rating of the E-interviews quality (*p* = 0.007) and the speed of the internet connection (*p* < 0.006), while their stress level with the E-interviews was independently and significantly associated with their preference for in-person interviews (*p* = 0.006). (Table 5).

**Table 5:** Multivariate logistic regression analysis of different factors and their association with the applicants’ odds of having stressful experience with E-interviews

	Adjusted odds ratio	95% CI for OR		***P***-value
		Lower	Upper	
Previously experienced with E-interviews (Yes)	2.351	1.221	4.526	0.011
Used E-interviews interface (FaceTime + Zoom)	3.264	1.296	8.221	0.012
Self-representation during the E-interviews (rating)	0.588	0.425	.813	0.001
Preferred E-interviews based on colleague’s advice	0.050	0.005	.540	0.014
Willingness to recommend E-interviews to others (rating)	1.213	0.920	1.600	0.172
Prefers E-interviews only (reference group)				0.012
Prefers both E-interviews and in-person	0.900	0.351	2.307	0.826
Prefers in-person interviews only	3.114	1.390	6.979	0.006
Perceived cost saving (rating scale)	0.386	0.282	0.529	< 0.001
Overall rating of the E-interviews quality (Likert rating scale)	0.557	0.365	0.850	0.007
Use of prior WhatsApp communication prior to the conference	1.630	0.845	3.144	0.145
Speedy Internet connection during the E-interviews	2.626	1.327	5.194	0.006
Not having a demonstration of the E-interviews application before the event	1.953	0.974	3.915	0.059
Overall rating of the E-interviews organization (Likert rating scale)	1.391	0.917	2.111	0.120
Constant	0.355			0.473

**Fig. 1:**
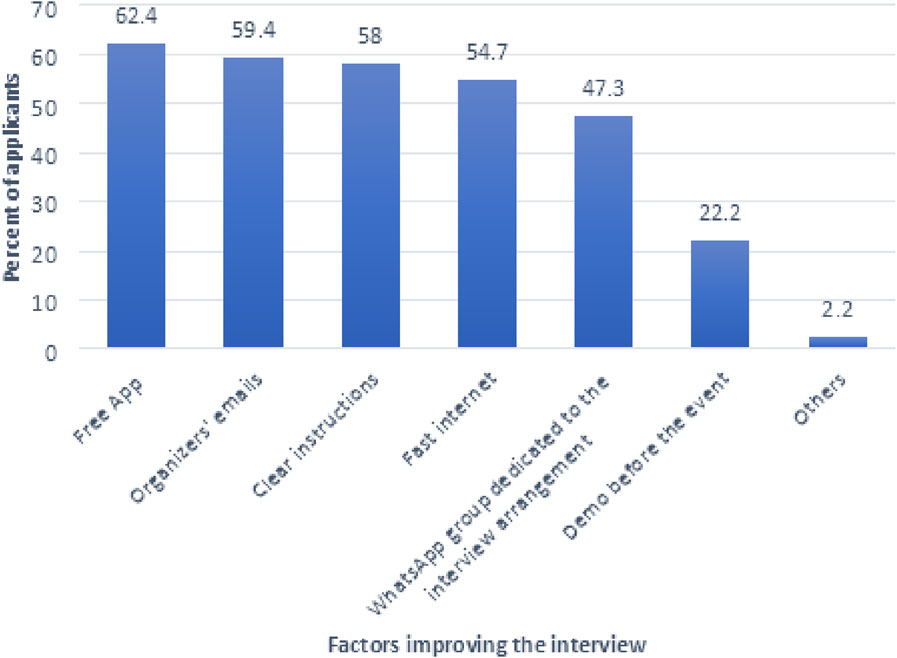
The medical residency candidates’ perceived factors that enhanced the evaluation by videoconferencing

When surveyed regarding different items that were perceived to contribute positively to the organization of the E-interviews, the top was free-use of the interviewing application by 62.4% of them, followed by the clarification emails they received from the organizers (59.4%) that made it a better experience, followed by the precise instructions given pre-event (58%) (Fig. 1).

**Fig. 2:**
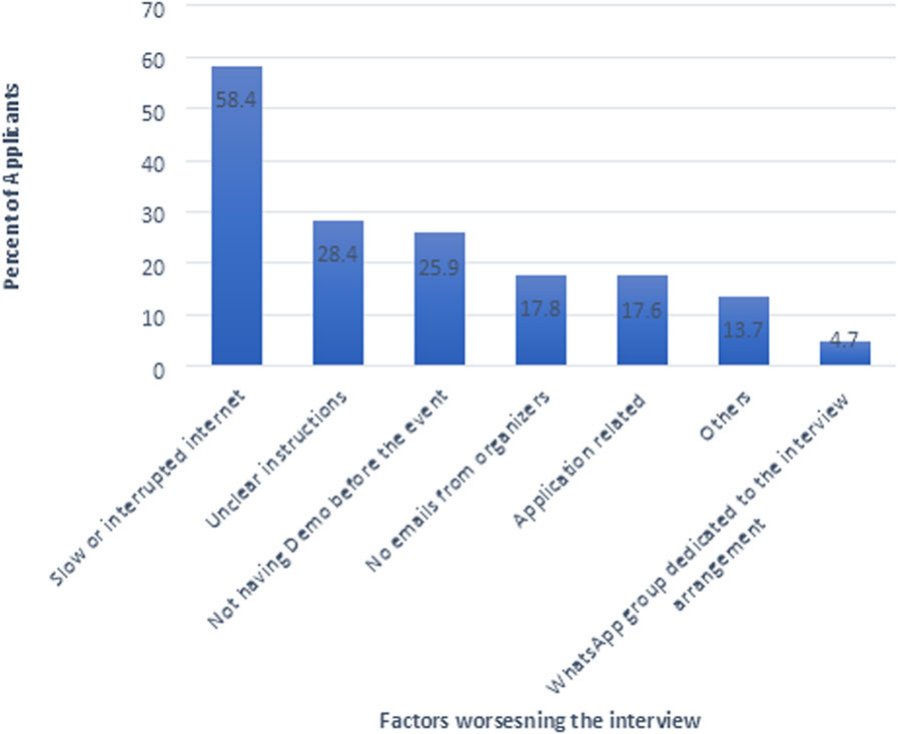
The medical residency candidates’ perceived factors that hindered the evaluation by videoconferencing

Conversely, the top perceived factors that negatively affected the E-interviews were the slow, inconsistent, and interrupted Internet speed and connection, the absence of clear instructions, and the lack of previous experience with these applications used for teleconferencing (Fig. 2).

## Discussion

In-person interviews were the primary interviewing method used for the postgraduate medical residency programs application process for decades. However, over the years, this method has faced some obstacles, including budget issues for the centers conducting multiple interview sessions for a large number of applicants on one side, and most applicants who were obligated to travel to other cities to attend these interviews admit that they are time- and money-consuming due to flights, accommodation, and transportation [1, 10]. In addition, these interviews are time-consuming for the applicants and the applied-to institutions, as well as their consequences on the clinical duties flow. Furthermore, in-person interviews require many efforts and preparation to schedule over multiple days with subsequent scheduling conflicts between centers, which could be hectic, especially when considering centers spreading over multiple cities across big countries [3].

W﻿ith the ever-emerging innovations and advancements in telecommunication, technology has become an evident influence in medicine [11, 12]. Besides, telemedicine is now being used to link major tertiary hospitals with peripheral primary care centers to provide adequate care, with increasing potential benefits of the innovative technology [13, 14]. Videoconferencing has been highly encouraged and mandated sometimes for conducting meetings and rounds in hospitals during the COVID pandemic [15]. E-interviews are not a new method of interviewing; some studies have compared in-person interviews with E-interviews in terms of financial costs, time consumption, effectiveness, and satisfaction. A study conducted in Washington, DC, USA, has shown that web-based interviews were cost-effective and time-saving for both applicants and residency programs [7]. Another study conducted at Kaplan Joint Center, Newton, Massachusetts, found that most study participants were satisfied with E-interviews [8].

Even though the advancement of information technology in our lives in general and in medical care as mentioned above, in our study, the applicants indicated that 61.2% never had the chance to use web-based videoconferencing before, and only 4.9% used it for E-interviews, although mobile phone use among medical residents was shown to have become almost universal in academic and clinical settings [16].

Our study has shown that 63.3% of the applicants preferred E-interviews over in-person interviews, and 60.6% rated their experience as very good to excellent. In a 2017 evaluation of web-based videoconference interviews for fellowship programs, 85% of the candidates felt satisfied [8].

When addressing the stress of E-interviews, which is the major psychosomatic focus for the applicant before, during, and after the interview, our survey found that 28.2% felt no stress at all, while 41.2% felt little stressed and only 8.2% felt highly stressed. A survey involving 400 residents in 2019 has found that 45% were stressed by their in-person interviews, which is much higher than that found in our study [17]. Stress level is highly linked to the ability of the applicants to present themselves. Although in-person interviews allow the interviewer to appreciate the applicant’s personality, reactions, attitude, and clinical sense more clearly, still, 52.2% of the applicants agreed that E-interviews allowed them to represent themselves accurately. On the other hand, 75% of the applicants felt that the E-interviews were adequate to answer all their queries. Finally, an inherited level of stress might be linked to the fact that stress is embedded in the process of application due to the competitive atmosphere and limited acceptance chances that is appreciated by applicants regardless to the interview format.

The financial investment needed for on-site interviews and the time spent distract both staff and applicants from their educational pursuits and clinical responsibilities. Applicants are usually required to fund their travel accommodation and requirements, adding additional financial burden to an already costly medical education. Medical residency program interviews require allocating considerable funds from both sides for meals, tours, staffing, and others. Comparing in-person interviews with E-interviews, 35.7% of the applicants in our study noted that each of them saved more than 500 SR ($133) without considering saving from the centers’ side. Additional cost-saving was one of the independent variables associated with high applicants’ satisfaction with E-interviews. A previous study of residency program web-based interview was less costly by a mean of $171 /applicant than in-person interviews [9]. The cost is even higher in other countries, with residency applicants traveling for a median of 3 weeks and spending approximately $4000 [1, 10]. Adding to that stress of travel and preparation time for several interviews in various locations may decrease the efficacy of the applicants’ presentation and the resultant surge in their stress level during the interviews [7].

About 80% of them felt that the time dedicated for the E-interviews was at least enough. Johnson et al. (2019) has proposed that in-person interviews allow for more conversation; however, they do not differ significantly from other interviewing methods [18]. Furthermore, as some remote interviews might be necessary for some situations, it will reduce the information obtained during the interview. Therefore, during a teleconference interview, the interviewee’s stress level might be reduced as there is less demand for more detailed information, as compared to the in-person interview.

Most published studies regarding web-based videoconference interviews did not report factors that enhanced applicants’ experience and satisfaction from a technical viewpoint, as they primarily focused on reporting its efficacy, relevance, and applicability. However, when we look to the commonly reported reasons for choosing E-interviews, for example, cost reduction [1, 5, 6], the use of free applications to conduct interviews would be a crucial factor in preferring this experience, and this is by far the top perceived factor by the respondents in our study.

A recent study on web-based videoconference interviews for surgical fellowship recruitment during the COVID-19 pandemic has reported that three out of 16 applicants underwent mock interviews to facilitate their experience [19]. Two of the three applicants (66.7%) found it helpful, whereas, among the respondents of this study, 22% perceived it as helpful. While our E-interviews were prepared and conducted in an urgent matter, due to the pandemic crisis, future events could benefit from structured preparatory material and training for the E-interviews. Providing applicants with adequate information about this type of interview was performed in other studies and probably eased the interview flow. Furthermore, technology testing and registering software accounts ahead of time were recommended by Aparna Joshi et al. to avoid any obstacles on the event day [20]. They also recommended hiring a technology assistant to intervene whenever needed. Orientation pertaining to cybersecurity, identity theft, teleconferencing fatigue and others are among other important aspects that need to be targeted in E-interview participants.

Many factors have hindered the E-interviews experience for applicants; the most encountered factor was a slow or interrupted Internet connection, followed by the absence of clear instructions and the lack of previous experience in teleconferencing, whereas others faced difficulties like not receiving clear emails from the program directors.

Studies have also demonstrated the same problems, especially Internet connection issues. Both interviewers and applicants have faced Internet connection problems that resulted in low audiovisual quality. This could be avoided in the future with pre-event testing and technical support services, as it could be attributed to the fact that many applicants may not own the appropriate technology required for these interviews, and their home settings may not be suitable to hold a professional videoconference interview [4].

Shah et al. have suggested preventing such problems by establishing a protocol for troubleshooting before the actual interview. They provided written instructions for establishing a software account a month before the videoconference interview, conducting a test call with the program coordinator to verify a successful connection during the preceding week, and offering faculty members who were unfamiliar with the technology a 5-min tutorial on the day of the interview [6].

While our study explores a newly implemented E-interview system for the national residency programs in the region, it has some limitations. The self-reported nature of surveys carries potential recall bias. Also, while the respondents were from the national residency candidates; however, we did not collect data on each individual applicant’s location to verify their geographical representation across Saudi Arabia.

## Conclusion

E-interviews were successfully implemented urgently during the COVID-19 pandemic in the medical residency application process in Saudi Arabia. The residency applicants preferred videoconference interviews, along with the cost savings and easier logistics to conduct the interviews from various locations. Future studies to enhance this experience are warranted.

## Supplementary Information

**Additional file 1.**

## Data Availability

All data in this study will be made available upon reasonable request by directly contacting Dr. Mohamad-Hani Temsah at mtemsah@ksu.edu.sa

## Abbreviations

COVID-19: Cronavirus disease 2019
E-interview: Electronic interview videoconferencing
SCFHS: Saudi Commission for Healthcare Specialties
WHO: World Health Organization
