# Supplementary material for: Remote interviews for medical residency selection during the initial COVID-19 crisis: a national survey
Source: BMC Med Educ. 2021 Aug 31;21:462. doi: 10.1186/s12909-021-02890-7 (PMC8405253; doi:10.1186/s12909-021-02890-7)
Supplement: Supplementary file 1 — Additional file 1. [file 12909_2021_2890_MOESM1_ESM.pdf]

Video-Conference Interviews in the Residency Application Process during COVID19 Pandemic

Residency Application Feedback March 2020 - IRB approval code 0420-03

Dear Colleague,

Thanks for your valuable participation in the Residency Application with SCFHS in March 2020.

We appreciate it if you can provide us your feedback on your videoconferencing in these interviews.

This is IRB approved proposal, that will also help us to optimize the videoconferencing for residents in the future.

This is an anonymous survey that will not be linked to you, and your voluntary participation can help us to further improve the process in the future.

You can also choose to participate in the lucky draw for online coupons.

Thanks and Be Safe!

Dr. Hani Temsah
mtemsah@ksu.edu.sa

Dr. Fadiah AlKhattabi
fkhatabi@kfshrc.edu.sa

Dr. Basim Alsaywid
drbasim@yahoo.com
SCFHS Research Team

* 1. **To start the 5 minutes survey, please choose:**

- ◯ I took part in the residency interviews of SCFHS residency application in 2020 and I agree to participate
- ◯ I do not agree to participate

Video-Conference Interviews in the Residency Application Process during COVID19 Pandemic

Demographics

* 2. You are:

- ◯ Faculty member
- ◯ Resident applicant
- ◯ Chief resident
- ◯ Coordinator

* 3. Prior to this Video Interview, did you have prior experience in this tool?

- ◯ No, my first time to use video conferencing
- ◯ Yes I used video conferencing before, but first time to use it for residency interview
- ◯ Yes I used video conferencing before, including for residency interview

* 4. The videoconferencing tool you used:

- ◯ PC (laptop)
- ◯ PC (Desk top)
- ◯ Mobile

* 5. What is your gender?

- ◯ Female
- ◯ Male

* 6. Your residency application:

- ◯ Pediatrics
- ◯ Family medicine
- ◯ Internal medicine
- ◯ Surgery
- ◯ Emergency medicine
- ◯ Psychiatry
- ◯ Other (please specify)

Video-Conference Interviews in the Residency Application Process during COVID19 Pandemic

Faculty page:

* 7. How much did you find the video interview, as compared to previous traditional face-to-face interviews:

	Strongly disagree	Disagree	Neither agree or disagree	Agree	Strongly agree
The interview allowed me to accurately get idea about the applicant's personality	◯	◯	◯	◯	◯
My questions for the candidates were answered	◯	◯	◯	◯	◯
I felt comfortable ranking the candidates based on my video interview	◯	◯	◯	◯	◯

* 8. Which video conferencing tool did you use?

- ◯ All through Face Time
- ◯ Mostly Face Time, Few Zoom
- ◯ Almost equal: Face Time and Zoom
- ◯ Other (please specify)
- ◯ Mostly Zoom, Few Face Time
- ◯ All through Zoom

* 9. What factor(s) made you prefer one video conferencing tool over the other? (Please choose all that apply)

- ☐ My own personal preference (example: previous experience with Zoom or Face Time)
- ☐ The candidates preference
- ☐ The ease of use (user-friendly) of the App
- ☐ Other (please specify)
- ☐ My colleague's advice
- ☐ My mobile operating system (Apple versus Android-based)

* 10. Number of your academic experience as consultant:

- ◯ less than 5 years
- ◯ 5 - 10 years
- ◯ more than 10 years

Video-Conference Interviews in the Residency Application Process during COVID19 Pandemic

Applicant's page:

* 11. Which video tool did you use in this interview:

- ◯ Face Time
- ◯ Zoom
- ◯ Other (please specify)

* 12. How much did you find the video interview, as compared to previous traditional face-to-face interviews:

	Strongly disagree	Disagree	Neither agree or disagree	Agree	Strongly agree
The interview allowed me to accurately represent who I am	◯	◯	◯	◯	◯
My questions about this residency program were answered	◯	◯	◯	◯	◯
I feel comfortable ranking hospitals based on my interview	◯	◯	◯	◯	◯

* 13. What factor(s) made you prefer one video conferencing tool over the other? (Please choose all that apply)

- ☐ My own personal preference (example: previous experience with Zoom or Face Time)
- ☐ The ease of use (user-friendly) of the App
- ☐ The committee preference
- ☐ Other (please specify)
- ☐ My colleague's advice
- ☐ My mobile operating system (Apple versus Android-based)

* 14. Approximately how much do you think this video interview (as compared to coming for face-to-face interview) saved you money in total (example: travel expenses, transportation, hotel accommodations...etc)

- ◯ Less than 100 SR
- ◯ 100-500 SR
- ◯ More than 500 SR

Video-Conference Interviews in the Residency Application Process during COVID19 Pandemic

Chief Resident or Coordinator page:

* 15. How much did you find the video interview, as compared to previous traditional face-to-face interviews:

	Strongly disagree	Disagree	Neither agree or disagree	Agree	Strongly agree
The interview allowed me to accurately get idea about the applicant's personality	○	○	○	○	○
My questions for the candidates were answered	○	○	○	○	○
I felt comfortable ranking the candidates based on my video interview	○	○	○	○	○

* 16. Which video conferencing tool did you use?

- ○ All through Face Time
- ○ Mostly Face Time, Few Zoom
- ○ Almost equal: Face Time and Zoom
- ○ Other (please specify)
- ○ Mostly Zoom, Few Face Time
- ○ All through Zoom

* 17. What factor(s) made you prefer one video conferencing tool over the other? (Please choose all that apply)

- ☐ My own personal preference (example: previous experience with Zoom or Face Time)
- ☐ The candidates preference
- ☐ The committee preference
- ☐ Other (please specify)
- ☐ The ease of use (user-friendly) of the App
- ☐ My colleague's advice
- ☐ My mobile operating system (Apple versus Android-based)

Video-Conference Interviews in the Residency Application Process during COVID19 Pandemic

Satisfaction with the interview process:

* 18. How likely is it that you would recommend the video conferencing interviews to a friend or colleague?

NOT AT ALL LIKELY | EXTREMELY LIKELY

0	1	2	3	4	5	6	7	8	9	10

* 19. Currently with the COVID19 Pandemic: How do you find Video Interviews as compared to face-to-face interviews?

- ◯ Video Interviews are preferable
- ◯ Equally preferable
- ◯ Face-to-face interview are preferable

* 20. How much do you agree with the following statements regarding this video interview:

	Strongly disagree	Disagree	Neither agree or disagree	Agree	Strongly agree
Doing remote video interviews during COVID19 pandemic decreased my anxiety	◯	◯	◯	◯	◯
Video conferencing interviews decreased the costs for the candidates	◯	◯	◯	◯	◯

21. Overall, how would you rate the event?

- ◯ Excellent
- ◯ Very good
- ◯ Good
- ◯ Fair
- ◯ Poor

* 22. What factors **improved** your video conferencing interview?
(please choose all that apply)

- [ ] organizers' communication
- [ ] WhatsApp group discussion that related specifically to this event
- [ ] emails from the organizers
- [ ] Demo of the video conferencing App before the event
- [ ] Other (please specify)
- [ ] clear instructions
- [ ] free application (Zoom or Face Time)
- [ ] fast internet speed

* 23. What factors **negatively affected** your video conferencing interview?
(please choose all that apply)

- [ ] WhatsApp group discussion that related specifically to this event
- [ ] not receiving the emails from the organizers
- [ ] Not having Demo of the video conferencing App before the event
- [ ] Other (please specify)
- [ ] unclear instructions
- [ ] application related (for example: unfamiliar with Zoom or Face Time)
- [ ] slow or interrupted internet speed

24. How organized was the event?

- Extremely organized
- Very organized
- Somewhat organized
- Not so organized
- Not at all organized

* 25. Prior to the event, how much of the information that you needed did you get?

- All of the information
- Most of the information
- Some of the information
- A little of the information
- None of the information

* 26. Was the event length too long too short or about right?

- Much too long
- Too long
- About right
- Too short
- Much too short

* 27. How much do you rate satisfaction with your videoconferencing in regards to:

	Extremely unsatisfied	Unsatisfied	Neither satisfied or unsatisfied	Satisfied	Extremely satisfied
Picture quality	○	○	○	○	○
Voice quality	○	○	○	○	○
Battery/power supply issues	○	○	○	○	○
Your time management flexibility	○	○	○	○	○
Your place (office/hospital/home) flexibility	○	○	○	○	○

28. Is there anything else you’d like to share about the event?

Video-Conference Interviews in the Residency Application Process during COVID19 Pandemic

* 29. Have you experienced any of the following symptoms immediately after your online video-interview experience?

	Never	Almost never	Sometimes	Fairly Often	Very Often
In the last month, how often have you been upset because of something that happened unexpectedly?	◯	◯	◯	◯	◯
In the last month, how often have you felt that you were unable to control the important things in your life?	◯	◯	◯	◯	◯
In the last month, how often have you felt nervous and “stressed”?	◯	◯	◯	◯	◯
In the last month, how often have you felt confident about your ability to handle your personal problems?	◯	◯	◯	◯	◯
In the last month, how often have you felt that things were going your way?	◯	◯	◯	◯	◯
In the last month, how often have you found that you could not cope with all the things that you had to do?	◯	◯	◯	◯	◯
In the last month, how often have you been able to control irritations in your life?	◯	◯	◯	◯	◯
In the last month, how often have you felt that you were on top of things?	◯	◯	◯	◯	◯
In the last month, how often have you been angered because of things that were outside of your control?	◯	◯	◯	◯	◯
In the last month, how often have you felt difficulties were piling up so high that you could not overcome them?	◯	◯	◯	◯	◯

30. **Optional question:**

If you want to join our lucky draw for online coupons, please provide your email (it will not be linked with your answers, as these will be confidential)
